# Diversity of Root-Associated Fungi of the Terrestrial Orchids *Gavilea lutea* and *Chloraea collicensis* in a Temperate Forest Soil of South-Central Chile

**DOI:** 10.3390/jof8080794

**Published:** 2022-07-29

**Authors:** Héctor Herrera, Tedy Sanhueza, Rafael Borges da Silva Valadares, Francisco Matus, Guillermo Pereira, Cristian Atala, María de la Luz Mora, Cesar Arriagada

**Affiliations:** 1 Laboratorio de Biorremediación, Departamento de Ciencias Forestales, Facultad de Ciencias Agropecuarias y Forestales, Universidad de La Frontera, Temuco 4780000, Chile; tedy.sanhueza@ufrontera.cl; 2 Programa de Magister en Manejo de Recursos Naturales, Universidad de La Frontera, Casilla 54-D, Francisco Salazar 01145, Temuco 4780000, Chile; 3 Instituto Tecnologico Vale, Rua Boaventura da Silva 955, Belém 66055-090, PA, Brazil; rafael.borges.valadares@itv.org; 4 Laboratory of Conservation and Dynamics of Volcanic Soils, Department of Chemical Sciences and Natural Resources, Universidad de La Frontera, Temuco 4780000, Chile; francisco.matus@ufrontera.cl; 5 Network for Extreme Environmental Research (NEXER), Universidad de La Frontera, Temuco 4780000, Chile; 6 Departamento de Ciencias y Tecnología Vegetal, Laboratorio Biotecnología de Hongos, Universidad de Concepción, Los Angeles 4440000, Chile; gpereira@udec.cl; 7 Instituto de Biología, Facultad de Ciencias, Pontificia Universidad Católica de Valparaíso, Valparaiso 2340000, Chile; cristian.atala@pucv.cl; 8 Scientific and Technological Bioresource Nucleus, Universidad de La Frontera, Temuco 4780000, Chile; mariluz.mora@ufrontera.cl

**Keywords:** mycoheterotrophy, Orchidaceae, orchid mycorrhizae, soil fungi, symbiosis

## Abstract

The diversity of orchid mycorrhizal fungi (OMF) and other beneficial root-associated fungi in temperate forests has scarcely been examined. This study aimed to analyze the diversity of mycorrhizal and rhizosphere-associated fungal communities in the terrestrial orchids *Gavilea lutea* and *Chloraea collicensis* growing in high-orchid-population-density areas in the piedmont of the Andes Cordillera with native forest (*Nothofagus-Araucaria*) and Coastal Cordillera with an exotic plantation (*Pinus-Eucalyptus*) in south-central Chile. We focused on rhizosphere-inhabiting and peloton-associated OMF in a native forest (Andes Cordillera) and a mixed forest (Coastal Cordillera). The native terrestrial orchids *G. lutea* and *C. collicensis* were localized, mycorrhizal root segments were taken to isolate peloton-associated OMF, and rhizosphere soil was taken to perform the metabarcoding approach. The results revealed that Basidiomycota and Ascomycota were the main rhizosphere-inhabiting fungal phyla, showing significant differences in the composition of fungal communities in both sites. *Sebacina* was the most-abundant OMF genera in the rhizosphere of *G. lutea* growing in the native forest soil. In contrast, *Thanatephorus* was the most abundant mycorrhizal taxa growing in the rhizosphere of orchids from the Coastal Cordillera. Besides, other OMF genera such as *Inocybe*, *Tomentella*, and *Mycena* were detected. The diversity of OMF in pelotons differed, being mainly related to *Ceratobasidium* sp. and *Tulasnella* sp. These results provide evidence of differences in OMF from pelotons and the rhizosphere soil in *G. lutea* growing in the Andes Cordillera and a selection of microbial communities in the rhizosphere of *C. collicensis* in the Coastal Cordillera. This raises questions about the efficiency of propagation strategies based only on mycorrhizal fungi obtained by culture-dependent methods, especially in orchids that depend on non-culturable taxa for seed germination and plantlet development.

## 1. Introduction

The widespread Orchidaceae family interacts with specific soil-inhabiting fungi, establishing in orchid mycorrhizal symbiosis. This symbiosis is essential in the initial developmental stages of most orchids, as their seeds are tiny (<1 mm), with insufficient reserves to sustain seed germination by themselves [1,2]. At this stage, compatible orchid mycorrhizal fungi (OMF) provide essential nutrients to the orchid embryo for seed germination and benefit the initial plantlet stage [3,4,5]. The nutrient transfer between orchids and fungi is considered an aggressive process in which hyphae are often decomposed by hydrolytic enzymes releasing nutrients (mycoheterotrophy) [6,7]. At plant maturity, some orchids maintain associations with the OMF active at the seed germination stage [8], whereas others can change mycorrhizal partners to other fungal taxa [9,10].

Terrestrial autotrophic orchids are often associated with mycorrhizal taxa from the Tulasnellaceae, Ceratobasidiaceae, and Serendipitaceae families [11]. Still, they can also interact with diverse phyla, including saprophytic, endophytic, and some commonly accepted phytopathogens [12,13,14]. The mycorrhizal fungus associated with an orchid usually depends on the species and is influenced by the orchid’s developmental stage, environmental conditions, and surrounding plants [15,16]. Therefore, knowing the diversity of fungal taxa inhabiting the orchid rhizosphere provides essential information to identify critical symbionts and fungi with crucial roles in plant growth, stress tolerance, and establishment. Furthermore, the dynamic of specific OMF and other beneficial fungal taxa in the plant rhizosphere can provide clues to understanding the ecology of fungal interactions with terrestrial orchids. Most of our understanding of the symbiotic associations of terrestrial orchids colonizing temperate forest ecosystems is from the northern hemisphere [17,18,19]. These studies have demonstrated the direct influence of the forest on the soil microbial communities and the preferences for specific mycorrhizal fungi [20,21,22]. Considerably less is known about terrestrial orchids, particularly those inhabiting the native temperate rainforest such as the Coastal Cordillera without volcanic influence and in the piedmont of Andes Cordillera with recent volcanic ash-derived soils in south-central Chile.

In the Andes Cordillera, many orchids are present, including epiphytic and terrestrial species [23,24]. In Chile, there are almost seventy species [25,26], which grow in grasslands or hilly sides in the native forest of the Andes and Coastal Cordillera and depend on specific OMF to germinate [3,27]. Such germination is complex given the particular requirements for mycorrhizal fungi, linked to enhanced climate change consequences and challenging climate conditions in summer (water shortage, high soil-surface temperature, and UV radiation) and winter (cold temperatures) [28,29,30], especially in the Andes Cordillera. In addition, recent studies have demonstrated that, although OMF can be isolated from mycorrhizal tissues, they have low efficacy in in vitro symbiotic germination tests, especially in orchids colonizing some sites in the Andes [3]. In orchids growing in near the Coast, germination seems to be more efficient, obtaining high germination rates in symbiotic germination tests [3]. Therefore, it is expected that different fungal taxa (probably non-culturable) are involved in this stage, and rhizospheric fungi with plant growth-promoting traits may also have key roles at early developmental stages.

The seeds of native terrestrial Andean orchids are minute (<1 mm), with thousands produced by each plant [3]. Such seeds must find compatible mycorrhizal fungi in the soil to provide the embryo with carbon and other nutrients to start the germination stage and activate the critical symbiosis for orchids throughout their lives [31]. Once such a symbiosis occurs, the metabolic interchange mainly benefits the orchid, despite recently being reported as a potential nitrogen source for the fungal partner [32]. Despite the successful isolation of OMF from the roots of native species and the verified ability of some isolates for seed germination [33,34], the distribution and presence of OMF and other beneficial root-associated microorganisms in the orchid rhizosphere are mainly unknown [35]. In some sites of the Andes and Coastal Cordillera, associated with native and exotic forests, large populations of orchids live sympatrically or allopatrically [3]. The symbiotic requirements that successfully promote seed germination and plantlet establishment are expected to occur in such populations. Such sites represent an opportunity to study and understand terrestrial orchids’ life cycle and ecology.

Recently, soil DNA metabarcoding has been applied to understand several ecological processes involving plants and microorganisms, including orchids’ fungal and bacterial associations [36,37,38]. Therefore, knowing the diversity of fungi in the rhizosphere of terrestrial orchids in sites with a high concentration may help unravel fundamental symbiosis for the conservation of endangered orchid populations. This study aimed to analyze the diversity of fungal communities inhabiting the rhizosphere of the terrestrial orchids *Gavilea lutea* (Pers.) M.N. Correa and *Chloraea collicensis* Kraenzl. growing in temperate forests in south-central Chile, as a way to initiate further studies on potential mycorrhizal and beneficial fungal partners in other sites. Here, two high-orchid-population sites were selected in the Andes and Coastal Cordillera at similar latitudes. As far as we know, this is the first study analyzing the diversity of fungal communities associated with the rhizosphere of temperate Andean orchids using a metabarcoding approach.

## 2. Materials and Methods

### 2.1. Sampling Site Description

The native terrestrial orchids were identified during the flowering season (December 2019) in two different sites with a high concentration of orchid individuals: (i) *G. lutea* that colonize a pristine forest’s understory on high-elevation slopes near Andes Cordillera (Table 1) and (ii) *C. collicensis* growing in a grassland site surrounded by exotic trees (*Pinus radiata* D. Don and *Eucalyptus globulus* Labill.) near a segment of the Nahuelbuta mountains in the Coastal Cordillera (Table 1). The soils in the Andes are classified as Andisol according to the USDA [39], with minor developments and intensely layered volcanic ash soils [40], and the soil from Coastal is from the Metrenco series, a member of the fine, mixed, mesic family of typic Paleudults (Ultisol) [41].

About 3 g of rhizosphere soil samples were taken from 4 random plants for each subsite (*n* = 3) to perform the three composite rhizosphere soil samples at each site (Andes and Coastal Cordillera). The roots were gently shaken, and the rhizosphere soil adhering to the roots was collected. Similarly, composite bulk soil samples were collected 3 m away from each sampled plant at a depth of 10 cm, with no presence of other orchid individuals. The soil samples were stored in ice, transported to the laboratory, and stored at −20 °C for further processing. A total of 400 g of sieved 2 mm topsoil samples (0–10 cm deep) was reserved for standard chemical analysis. Chemical characterization was conducted for soil pH (2.5:1 water: soil ratio) and soil organic matter by the Walkley and Black method [42]. Other soil properties such as cation exchange capacity, Al, Fe, N, P, and K available were obtained [43].

### 2.2. DNA Isolation and Processing

Total DNA from rhizosphere and bulk samples was extracted from 300 mg of soil using the Soil DNA Extraction Kit (Metagenom Bio Inc, Waterloo, ON, Canada) according to the manufacturer’s recommendations, and the DNA concentration was checked in a Qubit^®^ 2.0 Fluorometer (Thermo Fisher Scientific, Waltham, MA, USA). The DNA samples were sequenced at Macrogen (Seoul, Korea), following the conditions reported in Fuentes, et al. [44], with modifications. The amplicon libraries were prepared according to the Illumina PCR Quantification Protocol Guide. The ITS2 region was amplified using the primer set ITS2_3F (5′ GCATCGATGAAGAACGCAGC 3′) and ITS2_4R (5′ TCCTCCGCTTATTGATATGC 3′) [44,45]. The size of PCR-enriched fragments was visualized on an Agilent Technologies 2100 Bioanalyzer using a DNA 1000 chip. The libraries were sequenced using a 2 × 300-bp paired-end run [MiSeq Reagent Kit, v. 3 (MS-102-3001)] on a MiSeq (Illumina, San Diego, CA, USA) instrument according to the manufacturer’s recommendations. The raw data obtained in this study were submitted to the NCBI Sequence Read Archive (http://trace.ncbi.nlm.nih.gov/Traces/sra/) accessed on 9 September 2021, under accession number PRJNA761864.

### 2.3. Sequence Analyses

The quality of the resulting raw reads was checked using FastQC v0.11.5 and analyzed using QIIME2 v2019.10. A quality filter step was performed using the DADA2 algorithm, wherein the barcodes were removed, and reads were truncated at 280 and 250 for forward and reverse, respectively. Paired-end reads were joined, and then a quality-aware correcting model that denoises and remove chimeras and residual PhiX reads was applied. Reads were dereplicated, and amplicon sequence variants (ASV) were estimated. The taxonomic assignment was performed using a naive Bayes classifier with UNITE database v7.2, and the samples were rarified at a depth of 97.700. Alpha diversity measurements for diversity and evenness were calculated using Shannon’s diversity index and Pielou’s evenness, respectively, with QUIIME2, and the resulting values were compared using the Kruskal–Wallis test. The trends of fungal sequences were visualized by a principal coordinate analysis (PCoA), and hierarchical clustering was constructed based on the Bray–Curtis dissimilarity matrix. Statistical differences between treatments were estimated by a permutational multivariate analysis of variance (PERMANOVA), with 999 random permutations on the Bray–Curtis dissimilarity matrix. Differences between microbial communities associated with the orchid rhizosphere were explored by linear discriminant analysis (LDA) effect size (LEfSe) using a Kruskal–Wallis followed by a Wilcoxon rank-sum test for pairwise comparison, with a *p*-value of 0.05 as cut-off and a linear discriminant analysis score of 3.0. Additionally, the Indicspecies package was used to analyze the strength and statistical significance of the results. Similarly, the samples’ shared and unique fungal sequences were calculated and visualized using the MicEco package with Venn diagrams. The predicted ecological roles of the fungal OTUs were assigned using FUNGuild v1.1 [46] in Python v3.8.2. Statistical analysis and data visualization were performed in R software v3.6.3 (R Core Team 2018; https://www.R-project.org; accessed on 1 December 2021).

### 2.4. Isolation of Peloton-Associated Mycorrhizal Fungi

Compatible mycorrhizal fungi were isolated from the mycorrhizal tissues of the selected plants (Section 2.1), characterized and identified following standard procedures [47]. Briefly, the roots of the selected orchids (*n* = 12 per sampling point) were screened for the presence of mycorrhizal structures. A segment of the colonized roots (brownish tissues) was excised using a sterile scalpel. The selected tissues (*n* = 10 per plant) were superficially sterilized using a 1:1:8 (ethanol 95%, sodium hypochlorite (2.5% active chlorine), and sterile deionized water) solution. Afterward, the segments were rinsed ten times with deionized sterilized water in a laminar flow cabinet. Then, the pelotons were excised from the roots and placed in a Petri dish containing potato dextrose agar supplemented with streptomycin at 100 mM. The Petri dishes were incubated in darkness at room temperature until the growth of fungi from a peloton was detected. The mycorrhizal fungi that showed the phenotypic characteristic of OMF were selected for molecular identification following standard procedures [48].

### 2.5. Symbiotic Germination Tests

Symbiotic seed-germination tests were performed using the OMF isolated from pelotons to evaluate the capability of the strains to induce seed germination in vitro. A standard protocol was performed to promote the germination of orchid seeds [47]. The seeds from one mature capsule from *G. lutea* and *C. collicensis* were superficially disinfected in ethanol 20% for one min and sodium hypochlorite 10% for two min, then five washes in sterile deionized water. Then, the seeds were suspended in 30 mL of sterile deionized water. Then, 300 µL of the suspension was dispersed in Petri dishes containing 25 mL of oatmeal agar (OMA plus streptomycin 100 mM). A fungal plug (ø 5 mm) from the mycorrhizal fungi isolated from the respective orchid (*G. lutea* or *C. collicensis*) was placed in the center of the plate containing OMA and incubated for two months in darkness at 25 ± 1 °C. The effect of fungi on seed germination after 60 days was evaluated [49], and the germination index was calculated [47]. Symbiotic seed germination was considered positive when the tested fungi induced the embryo swelling (partially covered or enlarged embryo without testa). Quantitative data were analyzed by ANOVA. If the *p*-value indicated significant differences between treatments (*p* < 0.05), post-hoc pairwise comparisons were performed using the SD of means and Tukey’s multiple range test. Statistical significance was set at *p* < 0.05 using R software (R Core Team 2018; https://www.R-project.org; accessed on 1 December 2021).

## 3. Results

### 3.1. Soil Characteristics

The soil chemical analyses showed differences between the Andes and the Coastal Cordillera soil. Specifically, total nitrogen, phosphorus, soil organic matter, aluminum saturation, iron, and extractable aluminum were higher in the Andes than in soils from the Coastal site (Table 2). Only total potassium, cation exchange capacity, and base saturation values were more elevated in soils from the Coastal site (Table 2).

### 3.2. Sequence Analysis

The sequence analysis showed 1,602,893 raw reads in the 12 input libraries, which were reduced to 1,354,116 ASV after quality filtering, which ranged from 97,765 to 125,611 and was, on average, 112,843 (Table S1).

**Figure 1:**
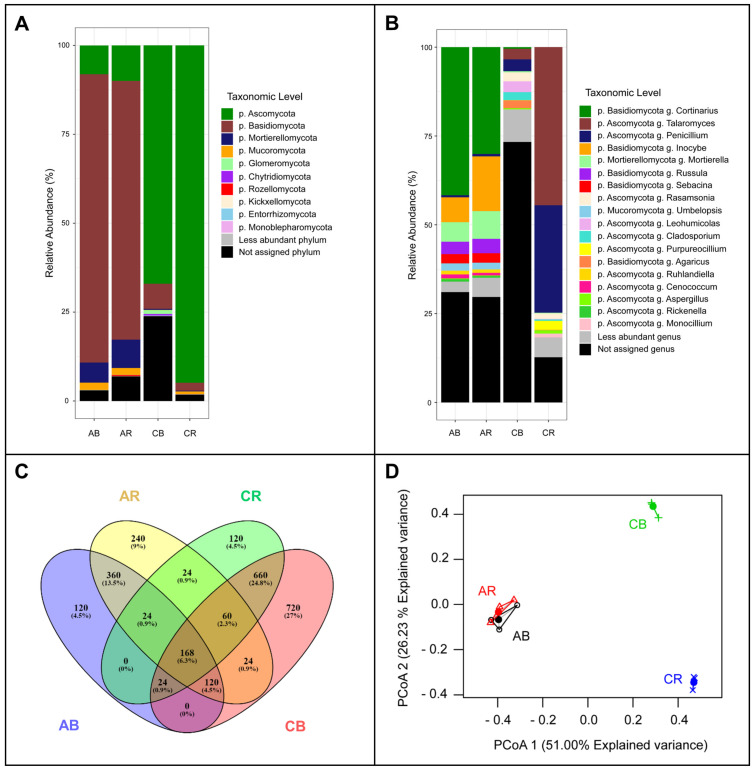
Fungal diversity associated with the rhizosphere of terrestrial orchids growing in the Andes (*Gavilea lutea*; AB: Andes bulk soil; AR Andes rhizosphere) and Coastal site (*Chloraea collicensis*; CB: Coastal bulk soil; CR: Coastal rhizosphere) in the region of La Araucanía, south-central Chile: (**A**) Relative abundance of ITS sequences at the phylum level, (**B**) relative abundance of ITS sequences at the genus level, (**C**) heatmap showing the specific and shared sequences between the sampling sites, (**D**) principal coordinate analysis of the fungal sequences from the analyzed sampling sites.

Shannon’s and Pielou’s evenness diversity indices were similar among the four analyzed samples, with higher values in bulk soil from orchids growing in the Coastal site (Figure S1). The taxonomic assignment showed that the fungal communities differed between sampling sites. The phyla Basidiomycota and Ascomycota were the most abundant in the Andes and Coastal Cordillera soil, respectively (Figure 1A; Table S2). Specifically, the genera *Cortinarius*, *Inocybe*, and *Mortierella* were among the most-abundant fungal taxa in both the rhizosphere and bulk soil of *G. lutea* plants growing in the Andes (Figure 1B; Table S3). In contrast, the rhizosphere and bulk soil of *C. collicensis* growing in the grassland site near the Coastal Cordillera showed differences among the identified genera. *Penicillium*, *Lechumicola*, and *Talaromyces* were the most-abundant assigned taxa in the bulk soil, whereas *Talaromyces*, *Penicillium*, and *Purpureocillium* were among the most-abundant fungal taxa in the rhizosphere (Figure 1B; Table S3).

The Venn diagrams showed 168 core sequences in the four samples, and samples from the Andes bulk soil, Andes rhizosphere, Coastal rhizosphere, and Coastal bulk soils have 120, 240, 120, and 720 unique sequences, respectively (Figure 1C). Similarly, the hierarchical clustering analysis showed that rhizosphere and bulk soil samples from the Andes were similar. By contrast, clear differences were detected between bulk soil and rhizosphere samples from the Coastal site (Figure 1D).

The LefSe analyses showed that there were 65 distinct fungal taxa at the genus level, with 42 differential taxa being associated with the rhizosphere of orchids from the Coastal site (mainly related to the phyla Ascomycota, Chytridiomycota, and Monoblepharomycota) and 23 fungal taxa associated with the rhizosphere of orchids from the Andes (mainly related to the phyla Basidiomycota, Mortierellomycota, Kickxellomycota, and Rosellomycota) (Figures S2 and S3).

**Figure 2:**
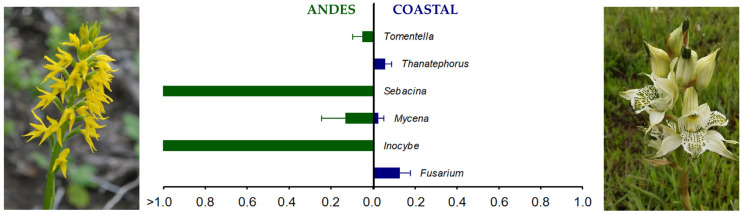
Relative abundance of the ITS sequences of different orchid mycorrhizal taxa in the rhizosphere of orchids growing in the Andes (*Gavilea lutea*) and Coastal Cordillera (*Chloraea collicensis*) in the region of La Araucanía, south-central Chile.

Regarding identifying fungal sequences assigned to previously reported OMF, we detected significant differences in the abundance of fungal ASVs in the rhizosphere of the analyzed plants. Specifically, *Tomentella*, *Sebacina*, and *Inocybe* were exclusively associated with *G. lutea* plants, showing a relative abundance of 0.05%, 2.64%, and 15.44%, respectively (Figure 2; Table S4). Similarly, *Thanatephorus* and *Fusarium* were identified in the rhizosphere of *C. collicensis* growing in the Coastal site, with a relative abundance of 0.06% and 0.13%, respectively (Figure 2; Table S4). Only *Mycena* was detected at both sampling sites, showing a relative abundance of 0.13% and 0.03% in the Coastal and Andes sites, respectively (Figure 2; Table S4).

The FUNGuild analysis showed that the categories symbiotroph and saprotroph_symbiotroph were most represented in soils from the Andes. By contrast, pathotroph, saprotroph, and pathotroph_saprotroph were the most represented categories in soils from the Coastal site (Figure S4).

A total of three strains with the phenotypic traits of OMF were isolated, two from *G. lutea* and one from *C. collicencis* (Genbank accession number (ON190562-ON190564). The isolation frequency of the OMF was higher in the isolate CCC2 (0.53), followed by GLM3 (0.42) and GLM5 (0.05). The molecular identification of culturable OMF showed *Ceratobasidum* spp. (isolates CCC2 and GLM3 from *C. collicencis* and *G. lutea*, respectively) as the primary mycorrhizal fungi isolated from both analyzed orchids, whereas *Tulasnella* sp. was scarcely isolated (isolate GLM5 from *G. lutea*) (Table 3). Symbiotic germination tests showed that the isolates have a contrasting capability to induce seed germination, showing a germination index of 0.75 ± 0.14, 0.58 ± 0.07, and 2.02 ± 0.54 for the isolates GLM3, GLM5, and CCC2, respectively (Table 3).

## 4. Discussion

This study used a metabarcoding approach to explore the diversity of mycorrhizal fungi associated with two native terrestrial orchids growing in the Andes and Coastal Cordillera in south-central Chile. Our data show differences in the composition and abundance of mycorrhizal taxa associated with terrestrial orchids growing in threatened temperate ecosystems in south-central Chile.

Soil-inhabiting fungi have been described as essential partners in the initial developmental stages of orchids and crucial in promoting seed germination and further establishment [50]. In this study, we have identified different fungal taxa associated with the orchid rhizosphere, including widely distributed soil-beneficial fungi such as *Cladosporium* spp., *Purpureocillium* spp., and *Mortierella* spp. These fungal taxa have been described as plant-growth-promoting fungi that contribute to the plant establishment in several ecosystems [51,52,53]. However, the roles of such fungi in terrestrial orchids have scarcely been explored. Recent studies analyzing the fungal communities associated with orchids have discovered beneficial fungi such as *Trechispora* and *Mortierella* inhabiting the rhizosphere of *Phalaenopsis* sp. [54]. Additionally, *Cortinarius* was the dominant taxon identified in the rhizosphere of *G. lutea* in the Andes (Figure 1B). The fungal genera *Cortinarius* include ectomycorrhizal fungi specialized in carbon mobilization through mycelial networks [55]. Correspondingly, the ectomycorrhizal genera *Cenococcum* was also detected in the rhizosphere of *G. lutea*. This is a persistent ectomycorrhizal fungus in dry forest soils and can remain active despite low water availability in the soil [56,57], such as in summer, the season in which the orchids were sampled. However, the possible specialization of orchids to this mycorrhizal fungus for carbon needs to be further studied. Beneficial rhizosphere-inhabiting fungi are critical to supporting the plant growth in severe ecosystems such as the Andes [58], where abiotic stress factors such as nutrient scarcity, extreme weather conditions (i.e., relatively dry summers), and the biochemical characteristics of the soil (i.e., presence of metals such as aluminum) can interfere with the growth of orchids in native ecosystems [3,59]. Thus, a set of beneficial fungi can contribute to the development of orchids through multiple mechanisms such as the promotion of abiotic stress tolerance, the production of plant growth regulators, the biocontrol of phytopathogens, the improvement of nutritional status, and others [60,61,62]. Therefore, studying rhizosphere-inhabiting fungi can provide insights into beneficial taxa contributing to the further developmental stages of terrestrial Andean orchids.

Our study results suggest a selection of root-associated fungal communities in *C. collicensis*, wherein a specific microbiome can be identified in the rhizosphere soil, contrary to the results of *G. lutea* in the Andes Cordillera (Figure 1B,D and Figure 2). These differences are related to the ecosystem characteristics, such as the presence of native or exotic species, chemical properties of the soil, and organic matter input, which underline specific changes in the microbiological communities but can also select particular taxa that can contribute to the life cycle of the associated plant [38,63,64]. This specialization was also detected in OMF independent of the sampling site, showing contrasting communities (Figure 2). Therefore, the dynamic of root-associated fungal communities in the analyzed population seems to depend on the surrounding environment and can have a critical role in the adaptation and fitness of terrestrial orchids in the ecosystem [65,66]. This issue must be considered when designing strategies for safeguarding endangered orchid populations.

Commonly, mycorrhizal fungi associated with orchids colonizing the southern Andes have been identified using culture-dependent methods [8], which provide essential information about orchid ecology and can help perform symbiotic germination tests under laboratory conditions. However, these assays are impossible when the orchid depends on mycorrhizal fungi that cannot be cultured using standard laboratory methods. This study demonstrated that the OMF isolated from pelotons differs from the main mycorrhizal taxa inhabiting the rhizosphere (Figure 2; Table 3). Previous studies have shown that some fungal strains directly isolated from pelotons could not induce seed germination and further progress to the plantlet stage under laboratory conditions [3]. Using the metabarcoding approach, we identify that the primary mycorrhizal fungus associated with the *G. lutea* rhizosphere belongs to Sebacinales, which have never been identified in any of the previous studies conducted in native terrestrial orchids colonizing temperate forest understory from Chile [3,27] but have been commonly reported in association with terrestrial orchids such as *Epidendrum* sp. growing in the Andes of southern Ecuador and in terrestrial Mediterranean orchids from the coast from Chile [38,67].

Although potential mycorrhizal strains were isolated from *G. lutea* roots (isolates GLM03 and GLM05), the ability to induce seed germination and advance to further stages was limited (compared to *C. collicensis*; Table 3). Different interactions with mycorrhizal fungi at the seed germination and plantlet stages can explain this low capability of advancing to further stages of seed germination. This agrees with Wang et al. [52], who showed that several orchids that transit from an initial mycoheterotrophic to a partially or fully autotrophic stage (which is present in terrestrial Andean orchids) often denotes a change in the fungal symbiont. Therefore, this change may explain the low efficiency of OMF isolated from pelotons to promote seed germination and further plantlet growth under laboratory conditions [9]. On the other hand, *C. collicensis* showed *Thanatephorus* spp. to be the main mycorrhizal taxa detected in the rhizosphere. Such OMF has previously been isolated from terrestrial orchids, but their ability to induce seed germination remains unclear [68]. In this study, the metabarcoding analysis did not detect *Ceratobasidium* sp., the most effective OMF for seed germination, colonizing the rhizosphere of the analyzed orchids. However, we identified *Ceratobasidium* sp. in the mycorrhizal tissues of *C. collicensis* (Table 3). Sequences designated as *Thanatephorus* and *Ceratobasidium* were usually clustered in one clade and may represent congeneric taxa in Ceratobasidiaceae in previous studies [69,70]. In this study, we found ITS2 ASVs assigned to *Thanatephorus* and the peloton-associated fungi (isolate CCC2) assigned to *Ceratobasidium*, which probably underline the fungal differences from mycorrhizal structures and rhizosphere of *C. collicensis*. However, this can also be related to the specificity of the selected PCR primers, and, therefore, different sequences/assignments can be obtained. Hence, further phylogenetic studies are necessary to ascertain the differences among fungi.

In the rhizosphere, compatible OMF is active [36,71], contributing to the population dynamic of orchids inhabiting an ecosystem. In this study, the metabarcoding approach has revealed fungal taxa commonly associated with terrestrial orchids colonizing temperate forests in Europe and North America but not described as mycorrhizae in native orchids from the southern Andes. This is related to the limited number of studies analyzing the fungal diversity in the rhizosphere of terrestrial orchids in temperate forests from the Andes. Additionally, most studies exploring the distribution of OMF in Andean orchids usually focus on identifying the traditionally accepted mycorrhizae, and culture-dependent methods might reduce the possibility of detecting novel taxa [3,38,72]. Fungal species from the genera *Mycena*, *Inocybe*, and *Tomentella* have been commonly reported with a critical role in the seed germination stage and seedling growth of orchids with different trophic modes. For instance, the litter-decaying fungi *Mycena* has been described in association with the mycoheterotrophic orchid *Gastrodia confusa* in temperate regions in Asia [22]. Similarly, the mycoheterotrophic orchid *Epipogium aphyllum* exclusively interacts with the ectomycorrhiza-forming genera *Inocybe* in the temperate areas of the Eurasian region [73]. The partially mycoheterotrophic orchid *Oreorchis indica* can also form mycorrhiza with the obligate ectomycorrhizal fungus *Tomentella* colonizing a sub-boreal forest understory [74]. On the other hand, in Australia, orchid species have been found to associate with typical orchid mycorrhizal fungi such as *Sebacina*, *Tulasnella*, and *Ceratobasidium* [75,76,77,78]. Overall, these studies demonstrate that terrestrial orchids inhabiting the forest understory are dynamic and can establish symbiosis with common soil-inhabiting taxa from which the obtained carbon is essential to complement the lack/inefficiency of photosynthesis in the shadow environment.

Some OMF active in the orchid’s rhizosphere are challenging to isolate and identify using culture-dependent methodologies, but a potential role in early growth stages can be expected. Thus, multiple detection methods may complement our understanding of the population dynamic of threatened orchid populations. Indeed, this study has revealed the presence of the genus *Fusarium* in the rhizosphere of *C. collicensis* growing in the Coastal site, which has been listed as a novel OMF promoting the seed germination of the autotrophic epiphytic orchid *Bletilla striata* and also inhabiting the mycorrhizal roots of the Mediterranean Chilean orchid *Bipinnula fimbriata* [14,38]. Therefore, limiting the study of mycorrhizal fungi associated with orchids to culture-dependent methods may shorten the understanding of OMF active in the orchid rhizosphere, restricting the establishment of plantlets derived from symbiotic germination tests. The environmental conditions and soil substrate’s particular characteristics have directly affected the composition of compatible mycorrhizal fungi associated with orchids and their germination success [27,79]. Therefore, multiple factors must be considered for the successful germination of orchids from Andean ecosystems.

The high specificity of symbiotic fungi may limit the success of reintroduction programs based on symbiotic or asymbiotic germination. Therefore, more than focusing on single strains, rhizosphere-associated community management strategies can be more effective at preserving threatened orchid populations, especially when the focus is on orchids that can interact with non-culturable mycorrhizal fungi. Hence, approaches involving compatible OMF and beneficial root-associated taxa must be implemented to manage endangered orchid populations better.

## 5. Conclusions

This study showed that the terrestrial orchids *G. lutea* and *C. collicensis* colonizing temperate forest soils in south-central Chile interact with different rhizosphere-associated and mycorrhizal fungal communities depending on the growing ecosystems. Similarly, the orchid mycorrhizal fungi associated with pelotons differed from mycorrhizal taxa inhabiting the rhizosphere in *G. lutea* from the Andes Cordillera. Further studies must address the capability of the native fungal microbiome associated with large-orchid-population-density areas to support the seed germination and plantlet establishment and determine if the selection of mycorrhizal fungi is a common characteristic of terrestrial Andean orchids from temperate forests.

## Figures and Tables

**Table 1:** Location and general description of the sampling sites.

Sites	Malalcahuello (Andes Cordillera Piedmont)	Cholchol (Coastal Cordillera Piedmont)
Coordinates	38°26′12″ S 71°33′01″ W	38°35′27.6″ S 72°54′57.9″ W
Elevation	1616 m.a.s.l.	29 m.a.s.l.
Mean annual precipitation	1225 mm (18.8 to 341 mm) ^a^	464 mm (1.9 to 118 mm) ^a^
Mean annual temperature	7.4 °C (3.5 to 16.5 °C) ^a^	12.7 °C (7 to 23 °C) ^a^
Vegetation details/understory	*Nothofagus dombeyii*, *Araucaria araucana*, *Nothofagus antarctica, Alstroemeria* spp.	*Pinus radiata*, *Eucalyptus globulus*, *Ulex europaeus*, and a mixture of native grasses
Soil order	Andisol	Ultisol
Forest	Native humid temperate rainforest	Exotic tree plantations surrounded by native grassland

^a^ low and high monthly temperatures in the sampling sites.

**Table 2:** Chemical characterization of soil associated with orchid hotspots in the Andes and Coastal site in south-central Chile. Results are means ± standard deviation.

	Andes	Coastal
N ^a^	16.75 ± 0.96	12.00 ± 1.41
P ^a^	29.00 ± 8.83	4.25 ± 0.50
K ^a^	81.13 ± 9.24	109.48 ± 43.54
pH ^b^	5.53 ± 0.17	5.62 ± 0.15
SOM	8.50 ± 0.58	4.25 ± 0.50
K ^d^	0.21 ± 0.02	0.28 ± 0.11
Na ^d^	0.04 ± 0.01	0.10 ± 0.01
Ca ^d^	2.11 ± 0.81	4.32 ± 0.48
Mg ^d^	0.45 ± 0.18	1.50 ± 0.19
Al ^d^	0.40 ± 0.18	0.52 ± 0.31
Al saturation ^c^	13.42 ± 7.39	7.95 ± 4.92
Cation exchange capacity ^d^	3.20 ± 0.95	6.71 ± 0.44
Base saturation ^d^	2.81 ± 1.01	6.19 ± 0.74
Fe ^a^	74.75 ± 13.84	30.00 ± 5.10
Al_extractable_^a^	722.75 ± 29.00	264.50 ± 17.00
Soil texture	Sandy loam	Clay loam

^a^ mg kg^−1^, ^b^ In H_2_O, ^c^ Soil organic matter (%), ^d^ C mol_(+)_ kg^−1^ soil.

**Table 3:** Peloton-associated mycorrhizal fungi isolated from a native temperate rainforest in south-central Chile and their effect on the seed germination of the source (*Chloraea collicensis* and *Gavilea lutea*).

Isolate	Close Relative (% Identity)	GenBank Accession (Close Relative)	Isolation Source (Close Relative)	Germination Index
**CCC2**	*Ceratobasidium* sp. (99%)	MK792996	*Chloraea gavilu*	2.02 ± 0.54
**GLM3**	*Ceratobasidiaceae* sp. (99%)	MK876128	*Bipinnula* sp.	0.75 ± 0.14
**GLM5**	*Tulasnella* sp. (100%)	MK793004	*Chloraea lechleri*	0.58 ± 0.07

## Data Availability

The raw data obtained in this study was submitted to the NCBI Sequence Read Archive (http://trace.ncbi.nlm.nih.gov/traces/sra/; accessed on 9 September 2021) under accession number PRJNA761864.

## Supplementary Materials

The following supporting information can be downloaded at: https://www.mdpi.com/article/10.3390/jof8080794/s1, Figure S1: Alpha diversity measurement distribution of the fungal microbiome associated with bulk and rhizosphere soil samples of native terrestrial orchids growing in the Andes and Coastal Cordillera in southern Chile. Shannon diversity index (A) and Pielou’s evenness (B). Samples AB = Andes bulk soil, AR = Andes rhizospheric soil, CB = Coastal bulk soil, and CR = Coastal rhizospheric soil. Figure S2: Linear discriminate analysis (LDA) of effect size (LEfSe) to identify the preferential taxa at the phylum level in rhizosphere soil associated with terrestrial orchids from the Andes and Coastal Cordillera in Southern Chile. Only taxa with an LDA score > 3.0 are shown. Figure S3: Linear discriminate analysis of effect size to identify preferential taxa at the genus level in the rhizosphere of the orchid from both analyzed sites. Only taxa with an LDA score > 3.0 are shown. Figure S4. Relative abundance of ITS sequences representing different trophic modes of fungi in the rhizosphere of orchids growing in the Andes and Coastal Cordillera in the region of La Araucanía, south-central Chile. Table S1: Trimming summary showing the number of initial reads, reads that pass quality control, number of sequences after merging them by their 3’ ends, and resulting non-chimeric ASV in soil samples associated with terrestrial-orchid hotspots in the southern Andes. Table S2: Mean relative abundance of the ten most-abundant fungal phylum identified in soil samples associated with terrestrial orchid-hotspots in the south Andes. Table S3: Mean relative abundance of the eighteen most-abundant fungal genera identified in soil samples associated with terrestrial-orchid hotspots in the southern Andes. Table S4: Relative abundance (%) of the commonly reported orchid mycorrhizal fungi inhabiting the rhizosphere and bulk soil samples of terrestrial orchids growing in the Andes and Coastal Cordillera in south-central Chile. Dataset S1: Taxonomy assignment of fungal ASVs in rhizosphere soil associated with terrestrial orchids from the Andes and Coastal Cordillera in southern Chile.
