# Supplementary material for: Diversity of Root-Associated Fungi of the Terrestrial Orchids Gavilea lutea and Chloraea collicensis in a Temperate Forest Soil of South-Central Chile
Source: J Fungi (Basel). 2022 Jul 29;8(8):794. doi: 10.3390/jof8080794 (PMC9409917; doi:10.3390/jof8080794)
Supplement: Supplementary file 1 [file jof-08-00794-s001.zip › jof-1766912-supplementary (v2).pdf]

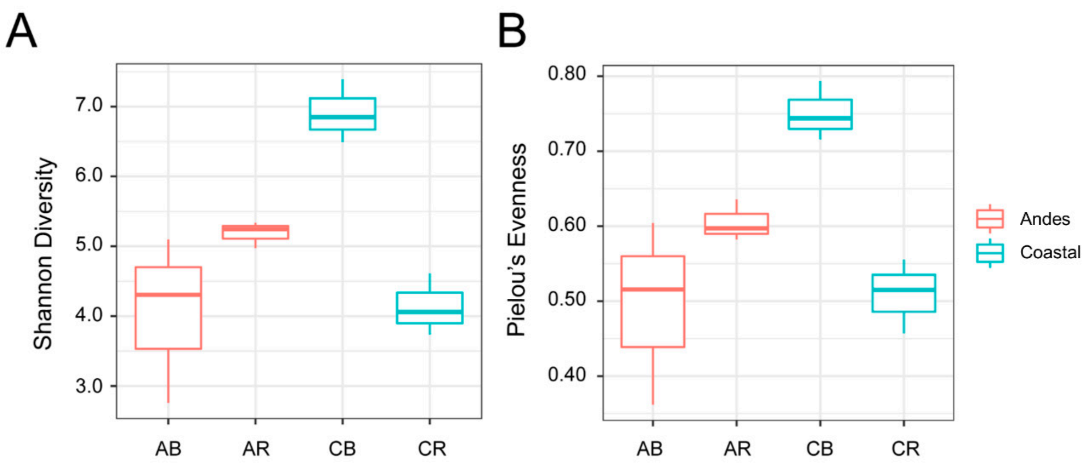

Figure S1. Alpha diversity measurement distribution of the fungal microbiome associated with bulk and rhizosphere soil samples of native terrestrial orchids growing in the Andes and Coastal mountains in southern Chile. Shannon diversity index (A) and Pielou's Evenness (B). Samples AB = Andes bulk soil; AR = Andes rhizospheric soil; CB = Coastal bulk soil; and CR = Coastal rhizospheric soil

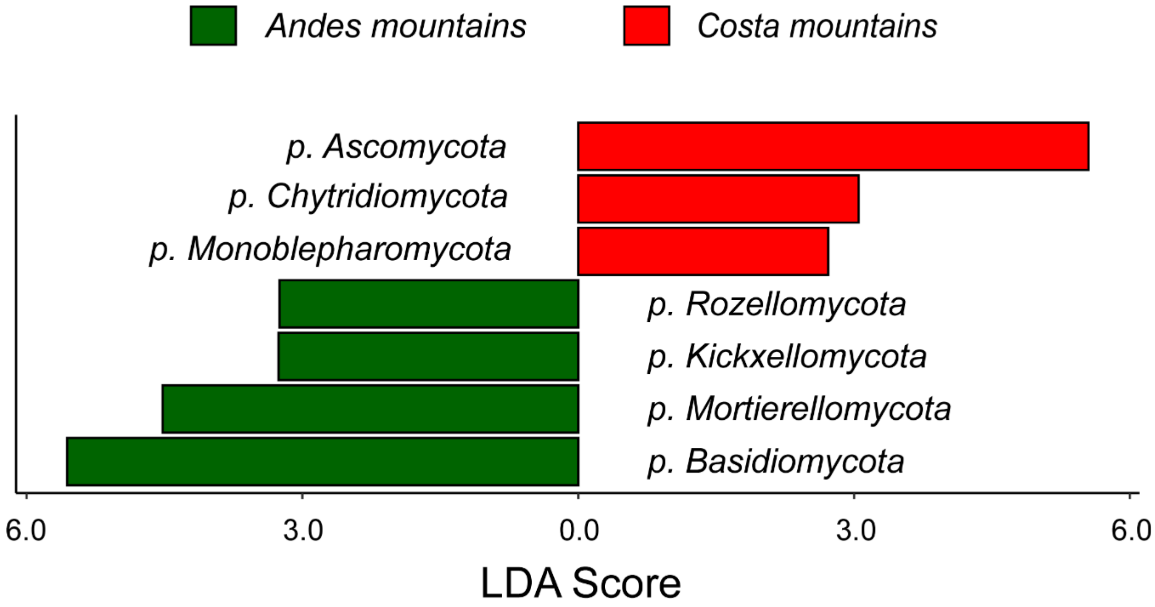

Figure S2. Linear discriminate analysis (LDA) of effect size (LEfSe) to identify preferential taxa at the phylum level in rhizosphere soil associated with terrestrial orchids from the Andes and Coastal mountains in Southern Chile. Only taxa with an LDA score > 3.0 are shown.

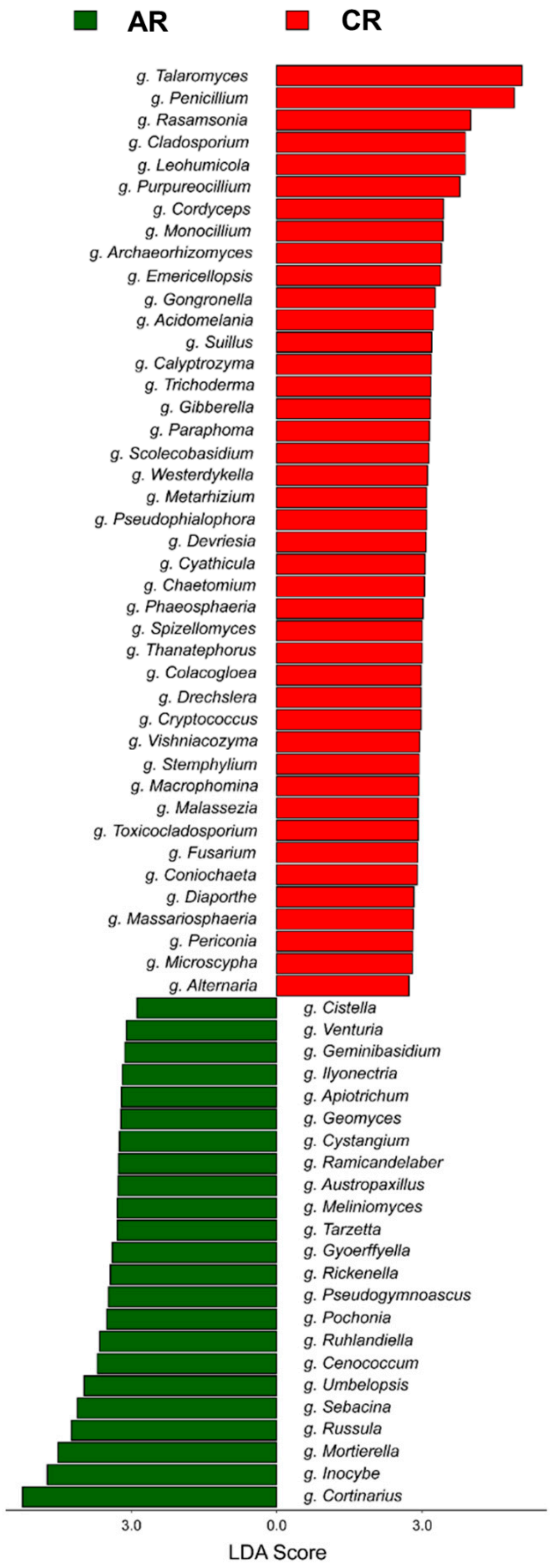

Figure S3: Linear discriminate analysis of effect size to identify preferential taxa at the genus level in the rhizosphere of the orchid from both analyzed sites. Only taxa with an LDA score > 3.0 are shown

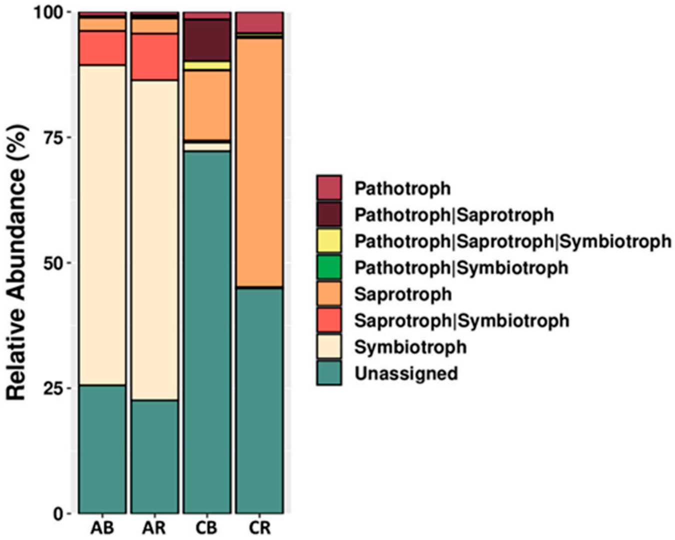

Figure S4. Relative abundance of ITS sequences representing different trophic modes of fungi in the rhizosphere of orchids growing in the Andes and Coastal Cordillera in the Region of La Ar-aucanía, south-central Chile

Table S1. Trimming summary showing the number of initial reads, reads that pass quality control, number of sequences after merging them by their 3' ends and resulting non-chimeric ASV in soil samples associated with terrestrial orchid-hotspots in the southern Andes.

Sample[1]	Raw Input	Denoised	Merged	Non-Chimeric
**AB-1**	134.048	120.987	120.445	118.565
**AB-2**	122.407	113.437	112.720	112.443
**AB-3**	133.316	121.782	120.374	119.867
**AR-1**	106.586	98.902	97.984	97.765
**AR-2**	124.946	115.773	113.529	112.955
**AR-3**	119.703	111.766	107.739	106.649
**CB-1**	129.956	114.995	112.032	110.136
**CB-2**	139.876	123.950	122.719	119.127
**CB-3**	121.540	107.783	106..487	104.002
**CR-1**	166.482	127.493	118.120	116.832
**CR-2**	163.125	134.008	128.577	125.611
**CR-3**	140.908	111.354	110.678	110.164

[1] Samples AB = Andes bulk soil; AR = Andes rhizospheric soil; CB = Coastal bulk soil; and CR = Coastal rhizospheric soil. The number 1, 2 or 3 refers to the biological replicates.

Table S2. Mean relative abundance of the ten most abundant fungal phylum identified in soil samples associated with terrestrial orchid-hotspots in the southern Andes.

**Taxa**	**AB**[1]	**AR**[1]	**CB**[1]	**CR**[1]
Ascomycota	8.07 ± 4.70	9.95 ± 5.23	67.04 ± 5.96	94.85 ± 1.14
Basidiomycota	81.09 ± 2.88	72.77 ± 8.20	7.04 ± 4.35	2.25 ± 0.92
Mortierellomycota	5.64 ± 0.82	7.97 ± 2.01	0.31 ± 0.19	0.20 ± 0.06
Mucoromycota	2.06 ± 2.48	1.94 ± 1.05	0.05 ± 0.02	0.79 ± 0.67
Glomeromycota	0.03 ± 0.03	0.04 ± 0.04	1.10 ± 0.39	0.05 ± 0.04
Chytridiomycota	0.00 ± 0.00	0.03 ± 0.03	0.42 ± 0.29	0.05 ± 0.04
Rozellomycota	0.00 ± 0.00	0.28 ± 0.45	0.00 ± 0.00	0.00 ± 0.00
Kickxellomycota	0.06 ± 0.07	0.08 ± 0.02	0.00 ± 0.00	0.00 ± 0.00
Entorrhizomycota	0.00 ± 0.00	0.00 ± 0.00	0.08 ± 0.09	0.00 ± 0.01
Monoblepharomycota	0.00 ± 0.00	0.00 ± 0.00	0.06 ± 0.02	0.02 ± 0.02

[1] Samples AB = Andes bulk soil; AR = Andes rhizospheric soil; CB = Coastal bulk soil; and CR = Coastal rhizospheric soil.

Table S3. Mean relative abundance of eighteen most abundant fungal genus identified in soil samples associated with terrestrial orchid-hotspots in the southern Andes.

Taxa	AB[1]	AR[1]	CB[1]	CR[1]
Cortinarius	41.68 ± 21.82	30.08 ± 17.85	0.44 ± 0.62	0.00 ± 0.00
Talaromyces	0.00 ± 0.00	0.00 ± 0.00	3.00 ± 2.05	44.50 ± 2.26
Penicillium	0.58 ± 0.18	0.66 ± 0.34	3.34 ± 0.95	30.25 ± 10.41
Inocybe	6.99 ± 8.16	15.42 ± 4.51	0.00 ± 0.00	0.00 ± 0.00
Mortierella	5.50 ± 0.68	7.81 ± 1.91	0.31 ± 0.19	0.19 ± 0.07
Russula	3.50 ± 3.13	4.05 ± 3.71	0.00 ± 0.01	0.00 ± 0.00
Sebacina	2.60 ± 1.74	2.64 ± 2.50	0.00 ± 0.00	0.00 ± 0.00
Rasamsonia	0.00 ± 0.00	0.00 ± 0.00	2.51 ± 0.90	1.52 ± 1.30
Umbelopsis	2.04 ± 2.48	1.90 ± 1.04	0.01 ± 0.02	0.02 ± 0.03
Leohumicola	0.03 ± 0.04	0.02 ± 0.03	3.06 ± 1.19	0.15 ± 0.10
Cladosporium	0.00 ± 0.00	0.00 ± 0.00	2.22 ± 2.78	0.42 ± 0.56
Purpureocillium	0.00 ± 0.00	0.00 ± 0.00	0.07 ± 0.04	2.52 ± 1.02
Agaricus	0.00 ± 0.00	0.00 ± 0.00	2.21 ± 3.82	0.13 ± 0.22
Ruhlandiella	1.04 ± 1.34	0.95 ± 0.86	0.00 ± 0.00	0.00 ± 0.00
Cenococcum	1.13 ± 0.93	0.74 ± 0.17	0.00 ± 0.00	0.00 ± 0.00
Aspergillus	0.22 ± 0.13	0.16 ± 0.02	0.35 ± 0.22	0.91 ± 0.71
Rickenella	0.67 ± 0.64	0.47 ± 0.65	0.00 ± 0.00	0.00 ± 0.00
Monocillium	0.00 ± 0.00	0.00 ± 0.00	0.03 ± 0.04	1.09 ± 1.87

[1] Samples AB = Andes bulk soil; AR = Andes rhizospheric soil; CB = Coastal bulk soil; and CR = Coastal rhizospheric soil.

Table S4. Relative abundance (%) of commonly reported orchid mycorrhizal fungi inhabiting the rhizosphere and bulk soil samples of terrestrial orchids growing in Andes and Coastal Cordilleras in south-central Chile

Taxa	AB[1]	AR[1]	CB[1]	CR[1]
Fusarium	0.00 ± 0.00	0.00 ± 0.00	0.09 ± 0.07	0.13 ± 0.09
Inocybe	6.99 ± 8.16	15.44 ± 4.52	0.00 ± 0.00	0.00 ± 0.00
Mycena	0.03 ± 0.02	0.13 ± 0.20	0.08 ± 0.13	0.03 ± 0.05
Sebacina	2.60 ± 1.74	2.64 ± 2.51	0.00 ± 0.00	0.00 ± 0.00
Thanatephorus	0.00 ± 0.00	0.00 ± 0.00	0.26 ± 0.40	0.06 ± 0.05
Waitea	0.00 ± 0.00	0.00 ± 0.00	0.03 ± 0.03	0.00 ± 0.00
Serendipita	0.00 ± 0.00	0.00 ± 0.00	0.02 ± 0.04	0.00 ± 0.00
Tomentella	0.04 ± 0.07	0.05 ± 0.08	0.00 ± 0.00	0.00 ± 0.00

[1] Samples AB = Andes bulk soil; AR = Andes rhizospheric soil; CB = Coastal bulk soil; and CR = Coastal rhizospheric soil.
